# A mixed-method feasibility study of a novel transitional regime of incremental haemodialysis: study design and protocol

**DOI:** 10.1007/s10157-021-02072-1

**Published:** 2021-06-08

**Authors:** Adil M. Hazara, Victoria Allgar, Maureen Twiddy, Sunil Bhandari

**Affiliations:** 1 Hull York Medical School, Hull, UK; 2 Hull University Teaching Hospitals NHS Trust, Anlaby Road, Hull, HU3 2JZ UK; 3 Peninsula Medical School, Faculty of Health, University of Plymouth, N15, ITTC Building 1, Plymouth Science Park, Plymouth, PL6 8BX UK; 4 Institute of Clinical and Applied Health Research, University of Hull, Hull, HU6 7RX UK

**Keywords:** Haemodialysis, Hemodialysis, Incremental HD, Chronic kidney disease, Dialysis

## Abstract

**Background:**

Incremental haemodialysis/haemodiafiltration (HD) may help reduce early mortality rates in patients starting HD. This mixed-method feasibility study aims to test the acceptability, tolerance and safety of a novel incremental HD regime, and to study its impact on parameters of patient wellbeing.

**Method:**

We aim to enrol 20 patients who will commence HD twice-weekly with progressive increases in duration and frequency, achieving conventional treatment times over 15 weeks (incremental group). Participants will be followed-up for 6 months and will undergo regular tests including urine collections, bio-impedance analyses and quality-of-life questionnaires. Semi-structured interviews will be conducted to explore patients’ prior expectations from HD, their motivations for participation and experiences of receiving incremental HD. For comparison of safety and indicators of dialysis adequacy, a cohort of 40 matched patients who previously received conventional HD will be constructed from local dialysis records (historical controls).

**Results:**

Data will be recorded on the numbers screened and proportions consented and completing the study (primary outcome). Incremental and conventional groups will be compared in terms of differences in blood pressure control, interdialytic weight changes, indicators of dialysis adequacy and differences in adverse and serious adverse events. In analyses restricted to incremental group, measurements of RRF, fluid load and quality-of-life during follow-up will be compared with baseline values. From patient interviews, a narrative description of key themes along with anonymised quotes will be presented.

**Conclusion:**

Results from this study will address a significant knowledge gap in the prescription HD therapy and inform the development novel future therapy regimens.

**Supplementary Information:**

The online version contains supplementary material available at 10.1007/s10157-021-02072-1.

## Introduction

In patients with advanced chronic kidney disease (CKD), the start of haemodialysis/haemodiafiltration (HD) therapy marks the start of a critical transitional period. The abrupt changes in patients’ lifestyles, independence and work [1, 2] combined with the additional burden of pathophysiological changes with the onset of regular dialysis [3–5] worsening indices of nutrition and inflammation with advancing CKD [6, 7] an increasing burden of co-morbid illnesses [8] and a decline in functional status especially in the elderly [9], can all lead to higher risk of decompensation in the early days of therapy [10, 11]. Mortality rates are highest in the first few weeks of treatment [12–14].

Incremental HD, which enables patients to start dialysis with smaller doses and gradually building up to traditional full dialysis over a longer period, may help reduce some of the associated risks early on by allowing patients the time to adapt physically and mentally to the new changes [15, 16]. Ultimately, a randomised control trial (RCT), testing incremental HD vs. standard care will be needed to definitively address if starting HD incrementally reduces mortality in incident dialysis starters [17, 18]. Key uncertainties need to be addressed, however, before a definitive RCT can be conducted:

A. Wide variations exist in the practice of incremental HD worldwide [19, 20]. The optimal design for a suitable regimen that is safe, effective and easy to administer needs to be fully established through feasibility testing before it can be formally tested in an RCT.
B. The level of patient interest and demand for incremental HD require further exploration. This information is needed for planning of resources in the future RCT.
C. The most suitable *primary* outcome measure for use in a future RCT needs further study (this information is needed for the sample size calculation)
D. Given the time constraints that come with the delivery of each dialysis session and the already high frequency of patient exposure to healthcare environment, which additional tests should be included in the future RCT to help fully understand the impact of incremental HD on patient’s health.

To pave the way for a future RCT of incremental HD vs standard care, a feasibility study is being conducted which aims to establish the acceptability, tolerance and safety of a novel incremental HD regime; and to explore the feasibility of data collection and follow-up of the participants.

## Materials and methods

### Study design

A mixed-method study with two key elements:

1. A prospective interventional study, with historical matched controls, involving new HD starters; testing the feasibility and safety of incremental HD and examining the impact of incremental HD on patients’ wellbeing (the ‘interventional cohort study’).
2. Semi-structured interviews of study participants to document and analyse the experiences of patients receiving incremental HD (the ‘interview phase’).

### Settings

A large University Teaching Hospital in the UK (Hull University Teaching Hospitals NHS Trust) and its three associated satellite dialysis units [21].

### Participants

Adults with CKD stage 5 (from any cause), commencing in-centre maintenance HD therapy for end-stage renal disease (ESRD) in the out-patient settings. Full eligibility and exclusion criteria are presented in Table 1. Pre-dialysis specialist nephrology input for at least 3 months is essential since those without full preparation for dialysis may require intensive dialysis at presentation [22, 23] (i.e., unsuitable for incremental HD).

**Table 1:** Eligibility criteria for study participants

Inclusion criteria	Exclusion criteria
Age ≥ 18 patients with CKD-5 who are about to start planned HD At least 3 months of prior specialist renal follow-up at the time of starting HD Able to meet all the study requirements Written signed informed consent	Age < 18 No prior contact with nephrologists for > 3 months Cross-over into HD from peritoneal dialysis Currently undergoing HD therapy Any condition which in the opinion of the investigator makes the participant unsuitable for entry into the study Participation in an interventional study in the preceding 6 weeks History of myocardial infarction in the preceding 3 months Inability to provide informed consent Inability to comply with the study schedule and follow-up

*CKD* chronic kidney disease, *HD* haemodialysis

### Recruitment

#### Recruitment for the intervention arm

Participants will be approached at the specialist pre-dialysis clinics or the dialysis unit with the participant information sheet (PIS). They will be given at least 24 h to consider the information. Following this, the investigator will meet patients in person to address their questions and obtain written informed consent if they show willingness to proceed. They will remain in-active in the study, and under follow-up with their usual care providers, until the decision is made to start HD by their caring physician. The timing of HD initiation will be made independently of the study investigators by the clinical care teams, based on clinical needs and in consultation with patients and their relatives. Clinicians will not be blind to patients’ participation in the study; some pre-dialysis CKD-5 patients may have already signed up for the study prior to commencing dialysis. Whether the knowledge of patients’ interest in incremental HD influences decisions about the timing of starting HD is not known. The starting eGFRs will be reported for both incremental and control arm patients which could help address this current knowledge gap.

#### Recruitment for control arm (historical controls)

Each participant in the incremental HD arm of the study will be matched with 2 patients who had previously received the conventional HD treatment (historical controls; matching ratio 1:2). Participants for the control arm will be identified using the local dialysis treatment database. All patients starting HD therapy since 2013, the inception date of the database, will be included in the searches. Their dialysis treatment data (including nursing notes and exceptions), along with details of their medical history will be extracted for further analysis (see below).

#### Matching method

Propensity scores matching (PSM) will be undertaken to match participants in the incremental HD group with controls. Propensity scores will first be computed using logistic regression analysis accounting for the following 14 variables: age, C-reactive protein (CRP), serum albumin, vascular access type, pre-dialysis serum creatinine, dialysis facility, body mass index (BMI), parathyroid hormone levels (PTH), diastolic blood pressure (BP), haemoglobin, systolic BP, vitamin K antagonist treatment, history of cardiovascular disease and antihypertensive treatment. These variables together predict 2-year mortality in new dialysis starters with sensitivity and specificity of 72% and 69% based on a study by Siga et al. [24]. Matching will take place using the nearest neighbour method (no pre-specified caliper range) with no replacements.

### The intervention (the incremental HD regime)

Participants in the intervention group will receive incremental HD using a novel approach, starting therapy on twice-weekly basis with progressive increases in the duration and frequency of sessions over 15 weeks, achieving conventional treatment times by the end of this period.

The treatment delivered will comprise of a ‘start’ phase, representing just the first 2 days of dialysis (this is similar to conventional start), followed by 3 incremental phases or steps (also see Fig. 2):

- Start (phase 1): (first 2 days of HD): day 1, 2-h HD session; day 2, 3-h HD session.
- Phase 2: twice-weekly 2-h long dialysis sessions for 2 weeks.
- Phase 3: twice-weekly 3-h long dialysis session for 6 weeks.
- Phase 4: three-times weekly 3-h long session for 6 weeks.

Following this, participants will continue long-term HD as per clinical requirements. The regime includes several safety elements including frequent fluid status monitoring (through clinical examinations and bio-impedance analyses) and regular potassium testing. Clinicians and dialysis nurses can override the regime at any point if clinically indicated.

The regime tested here has been developed by the study investigators (AMH, SB) in consultation with patient groups as a pragmatic way of reducing exposure to dialysis during the critical early transitional period. At the designing stage, patient engagement involved discussions with National Institute of Health Research (NIHR) patient research ambassadors for renal medicine at our centre and the members of Hull Kidney Patients Association (HKPA). In addition, a brief questionnaire was given out to 20 current HD patients to gauge their opinions on the current proposals. Patient feedback was very positive; patients particularly appreciated the focus on maintaining independence at the start of HD which they valued highly. HKPA members saw this as a much-needed advancement in dialysis therapies and felt that patients will be motivated to help. The group gave positive suggestions on how to narrow down the process of identifying potential participants e.g., by prioritising the sub-set of patients who were being referred for vascular access procedure (as they are the most likely patients to start dialysis soon). In the survey of 20 HD patients, 10 patients responded 7 of whom indicated that they would have taken part in the study if they had the opportunity.

### Sample size

As this is a feasibility study, a formal sample size calculation has not been performed. The recruitment target is 20 participants in the active interventional arm and 40 in the historical control group (1:2 matching).

### Follow-up

**Fig. 1:**
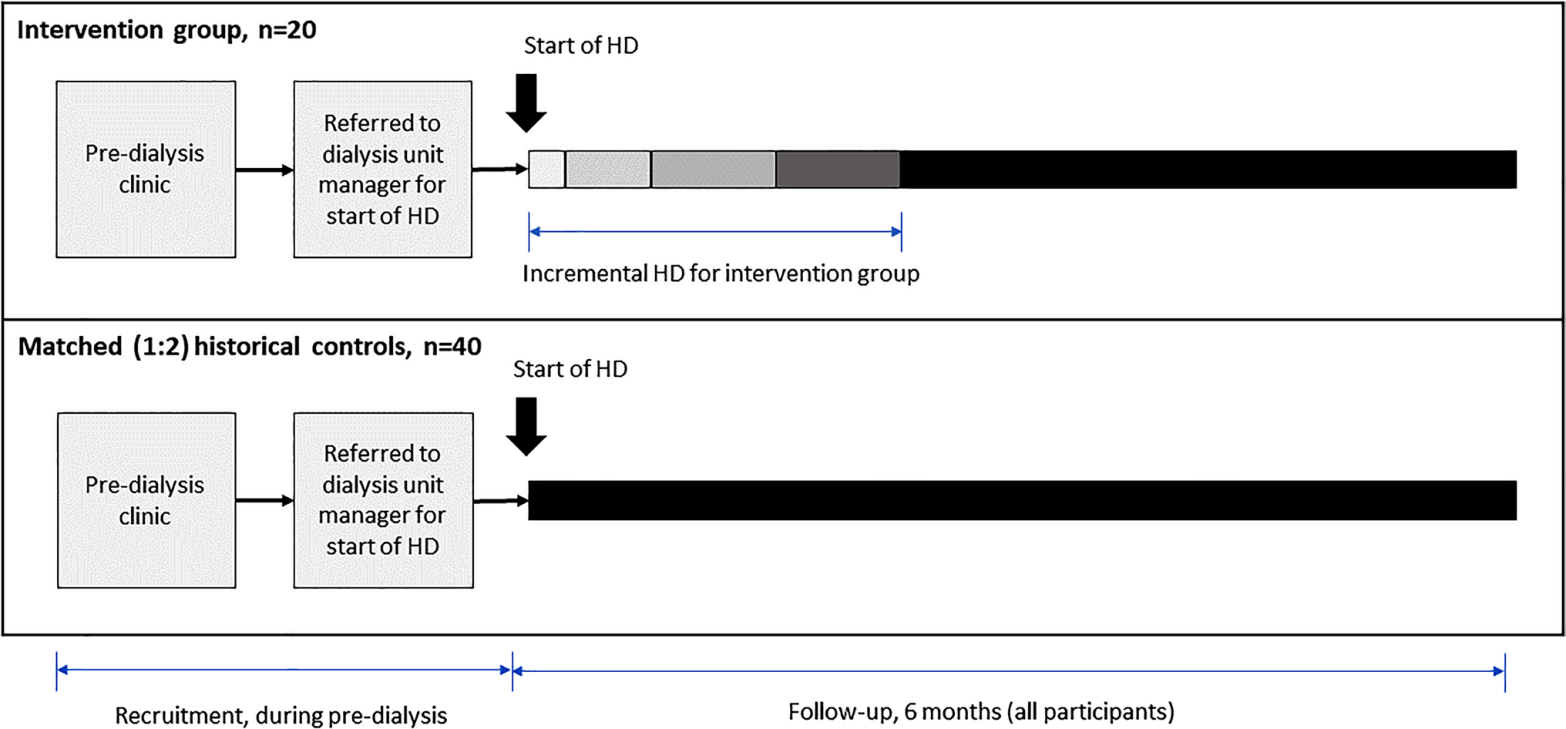
Recruitment and follow-up of patients in the intervention and control groups

Participants will be followed-up for 6 months from the start date of HD therapy (see Fig. 1 above). A total of eight study visits will be carried out for each participant as per the schedule presented in supplementary table S1. Participant monitoring, and data collection is more intense in the first 2 weeks.

### Measurements and data items

#### Baseline data

Data items recorded at baseline include age, gender, primary renal disease, names of co-morbid conditions, medications list (including the names of anti-hypertensives and diuretics), date of first contact with nephrology services, type of vascular access and estimated glomerular filtration rate (eGFR) based on serum creatinine measurement prior to first dialysis session. Charlson co-morbidity index [25] will be calculated for all participants to enable baseline comparison of the burden of co-morbidities. With patients’ permission, we will cross-reference medication lists with their primary care records to ensure completion.

#### Weights and blood pressures

Weight and blood pressure (BP) readings will be monitored at baseline and then at each subsequent dialysis visit. These will be measured pre- and post-dialysis. Inter-dialytic weight gain (IDWG) or loss will be recorded. BP readings taken during dialysis (intra-dialytic BPs) will also be recorded.

#### Blood tests

In routine dialysis practice, the drawing of ‘monthly bloods’[26] involve regular measurements of full-blood count (FBC), serum biochemical profile (BCP) and C-reactive protein (CRP), and 3-monthly measurements of serum Ferritin, Transferrin Saturations (TSAT) and Parathyroid hormone (PTH) levels. We will utilise these routine tests when making selected comparisons between patients in the two treatment arms (see outcomes). Bloods samples are drawn at the first dialysis session of each calendar month. Urea reduction ration (URR) and eKt/V_urea_ are calculated monthly using the pre- and post-dialysis serum urea levels [27].

Additional (non-routine) pre-dialysis BCP testing will be carried out on days 7, 10 and 14 of dialysis to ensure close monitoring of serum potassium levels during the first 2 weeks of treatment.

#### Urine collections and estimation of RRF

Serial 24-h interdialytic urine collections will be used to monitor RRF. Proportion of patients completing 24-h urine collections and urine volumes will be recorded. Renal urea clearance (KRU) will be estimated from the rate of urea excretion and time-averaged serum urea concentration change during the collecting interval using method described by Obi et al. [28].

Cystatin C levels will be measured pre-dialysis at baseline, 1, 3 and 6 months. There is controversy on whether Cystatin C alone adequately predicts GFR (or RRF) in *established* dialysis patients [29]. Its role in estimating GFR in new HD starters remains unexplored and the current study presents a unique opportunity to correlate RRF estimates from Cystatin C levels and urine collections over the first 6 months of dialysis.

#### Bio-impedance spectroscopy and fluid load

Bio-impedance spectroscopy (BIS) will be performed using Body Composition Monitor (Fresenius Medical Care, Germany) [30, 31]. Readings will be taken immediately pre-dialysis with patients reclined at 30 degrees [32] and electrodes placed on patient’s arm and foot on the same side of the body avoiding the vascular access side [33]. N-terminal pro-Brain type natriuretic peptide (NT-proBNP) levels will be measured at regular intervals to evaluate the impact of incremental HD on cardiac load [34, 35].

#### Quality of life and functional status

Quality of Life will be measured using the Kidney Disease Quality of Life Instrument-Short form (KDQOL-SF)™ v1.3 questionnaire [36] and performance status using the Karnofsky performance index [37, 38]. These will be performed at baseline and then repeated at 3 and 6 months. All tests, except 24-h urine collections, will be completed within the designated dialysis slot and will not require additional hospital visits or prolongation of hospital visits.

#### Safety and adverse events data

There are several safety measures built-in to our incremental HD program to protect patients from the deleterious effects of a rapid decline in RRF around the time of dialysis initiation or when at least three-times weekly dialysis is needed from the start. First, we are only recruiting patient with planned out-patient dialysis start; this excludes the majority of those who develop critical or life-threatening issues related to CKD-5. Furthermore, all participants are reviewed at the start (see study procedures in supplementary table S1) to ensure safety before patients commence the program. During the program, patients receive regular visits from the study team (in the first month, five study related checks are undertaken including review by the study medic on four occasions). Also, clinicians can change the dialysis schedule e.g., offer extra dialysis or to take the patient off the program completely at any point during the study; for this reason, the mid-week dialysis slot is always kept open in case a participant needs additional dialysis. Finally, this a short incremental regime lasting just over 3 months; hence, a decline in RRF is anticipated and treatment increases are pre-emptive in this program.

Information related to adverse events (AEs) and serious adverse events (SAEs) will be recorded continuously throughout the study based on definitions presented in supplementary table S2. Routinely, all dialysis treatments are recorded in the local dialysis treatment database and a named nurse documents a narrative account of that day’s treatment making notes of any significant events. This information will be accessed by the study team and interrogated for AEs and SAEs and substantiated with information documented elsewhere in patients’ electronic health records. Compliance with the programme and adherence (or deviations) to the prescription will be monitored.

Intra-dialytic hypotension is common and affects 17% of all dialysis sessions [39]. We define IDH in accordance with the European Best Practice Guidelines [40] as a symptomatic drop in BP during dialysis of ≥ 20 mmHg in systolic or ≥ 10 mmHg in mean arterial pressures, requiring nursing interventions such as the stopping of ultrafiltration, administration of saline or moving patients to Trendelenburg position. IDH events are recorded in the nursing entries as above which will be used as a basis for coding this information.

### Primary outcome

The primary outcome of this study is recruitment. We aim to recruit 20 patients over 18 months. Data will be kept on the number screened, proportion eligible, proportion consenting and proportion completing the study.

#### Justification for primary outcome measure

Ultimately, the aim of this feasibility is to pave the way for a future RCT. In the planning of such a RCT, estimates of patient recruitment and adherence ratios are necessary as it affects resource allocation [41]. We would consider an acceptance ratio of ≥ 40% (i.e., the proportion of patients enrolled from all those eligible and approached for the study) and retention rate of ≥ 70% (i.e., participants completing the study) to indicate feasibility. Additional HD sessions for clinical needs do not affect the retention rate as these are considered part of therapy regime along with other key safety indicators.

### Secondary outcomes

The secondary outcomes of this study are grouped in to following sub-categories:

A. Safety outcomes

To compare key indicators of patient wellbeing in those receiving incremental HD and conventional care over the 6-month follow-up period:

1. Differences in mean pre-dialysis BPs and IDWG (or loss)
2. Comparison of pre-dialysis potassium levels, serum albumin, PTH, adjusted calcium and phosphate levels.
3. Numbers of AEs and SAEs (based on definitions in table S2)
4. Numbers of hospitalisations
5. Numbers and rates of major adverse cardiovascular events (the 4p-MACE: a composite of cardiovascular deaths, nonfatal myocardial infarctions, nonfatal strokes and hospitalisations for unstable angina) [42]
6. Numbers and rates of all-cause mortality
7. Anaemia management: differences in Hb, Ferritin, TSAT and doses of IV iron and erythropoietin stimulating agent (ESA.)

B. Mechanistic outcomes

To study changes in RRF, fluid load, performance status and quality of life over 6 months in patients receiving incremental HD (incremental HD group only). All these are relative to baseline:

1. Change in RRF using urine volume, KRU and Cystatin C levels
2. Change in pre-dialysis fluid load as measured using BIS and Ntpro-BNP
3. Change in KDQOL-SF v1.3 scores (including in its sub-domains)
4. Change in Karnofsky performance index

C. Qualitative outcomes

To explore patients’ experiences of starting HD incrementally and of participating in the current study using semi-structured interviews—see interview sub-study section.

D. Further feasibility outcomes

To acquire data needed for planning a future RCT:

1. Progression to HD: some patients recruited for the current study may not start HD within the study period (the timing of onset of dialysis can be unpredictable). Of the patients recruited from pre-dialysis clinics, the proportion of patients progressing to receiving incremental HD within the study period will be reported. This will inform the number of additional participants needed in a future RCT and the duration of recruitment period.
2. Completion rates of the non-routine tests namely the 24-h urine collections, BIS and the KDQOL-SF v1.3 questionnaire.

### Analysis

The findings will be reported in accordance with the Consort Statement for Pilot and Feasibility trials [43]. A consort flowchart showing the number of patients screened, approached for participation, consented and those who eventually started treatment will be presented.

**Fig. 2:**
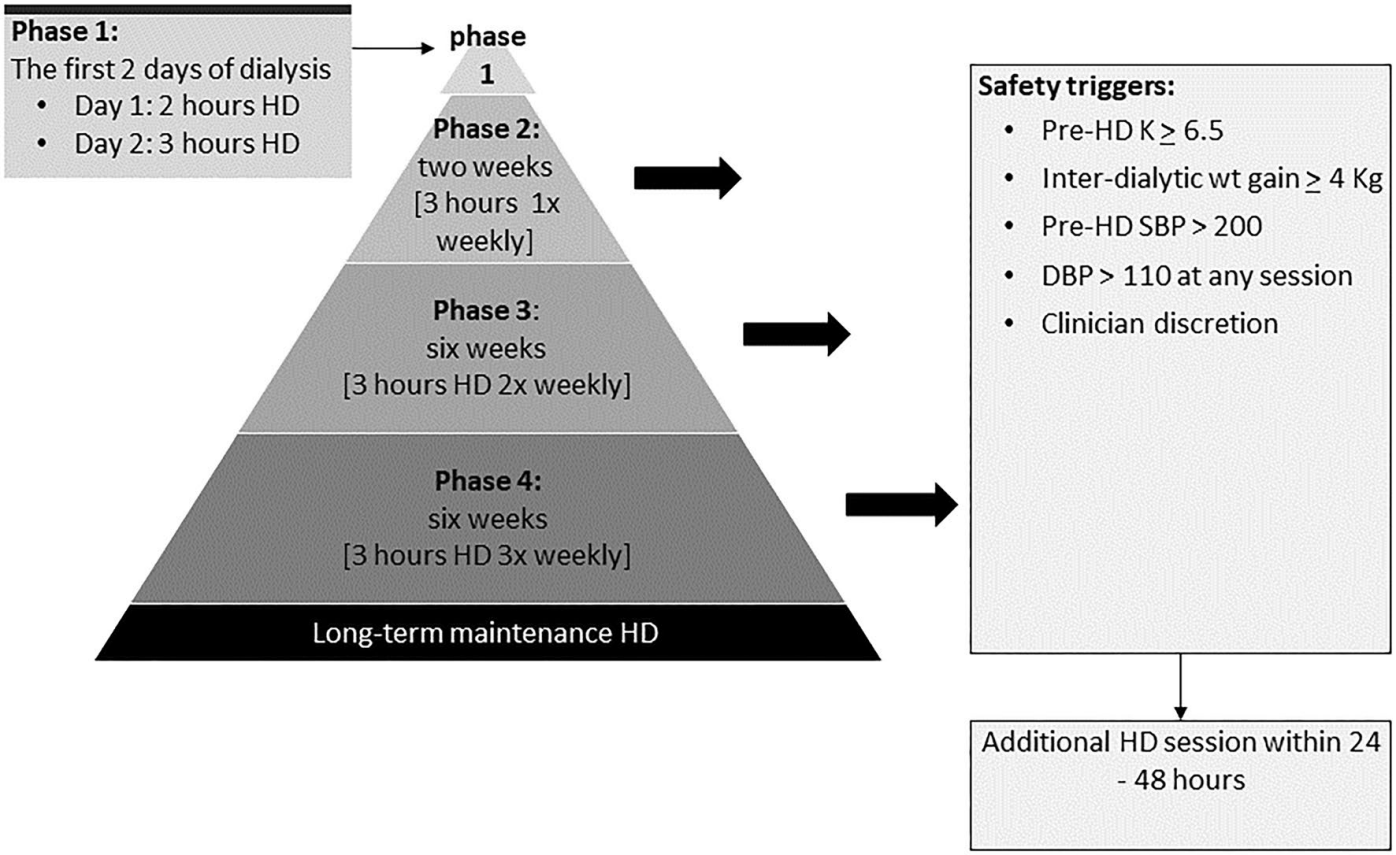
Incremental HD regimen used in the study showing the incremental steps

Patient follow-up period will be split in to the four phases of incremental HD regime (see Fig. 2 for phases of incremental HD). Details of dialysis treatments delivered in each phase to participants in both treatment arms will be expressed in sessions/week (frequency), minutes/week (duration), mean monthly URR and median sessional eKt/V_urea_ as well as OCM Kt/V_urea_. Given that patients in conventional HD group (historical controls) will have had more BPs and weight measurements recorded since they were on three-times weekly dialysis from the outset, their mid-week BP and weight measurements will not be used in computing these averages (to avoid regression towards mean). Weights, BPs and IDGW will be summarised as means (and SDs) for each treatment phase.

Results of routine tests (including Hb, potassium, albumin, PTH, adjusted calcium, phosphate), and IV iron and ESA doses administered will be summarised (as mean and SD or median and inter-quartile range as appropriate) for each treatment phase. For patients in incremental HD group, urine volumes, KRU, serum Cystatin C levels, pre-dialysis overhydration measured through BIS (expressed in millilitres), NT-pro-BNP levels will be summarised as means or medians as appropriate for each measured time point (baseline, 1-, 3- and 6-month). Differences in these measurements over the follow-up period will be compared to their baseline values.

Numbers of deaths from all causes, hospitalisations, the 4-point major adverse cardiovascular events (4p-MACE: a composite of cardiovascular deaths, nonfatal myocardial infarctions, nonfatal strokes and hospitalisations for unstable angina), AEs and SAEs will be reported for both the treatment arms.

Formal statistical tests will not be undertaken on secondary outcomes due to the feasibility design of this study (descriptive comparisons will, however, be drawn to examine findings for potential signals).

### Semi-structured patient interviews

Although there are theoretical advantages in reducing the burden of treatment at the start (in incremental HD), gold-standard evidence for its effectiveness is currently lacking. In this context, participants who agree to receive incremental HD hold a unique perspective that could aid, and enhance the design of a future RCT.

#### Aims of patient interviews

The key aims of patient interviews are to:

- Capture patients’ prior expectations and understandings of starting dialysis treatment
- Elicit their experiences of being approached for participation in the study
- Understand their motivation for consenting to a trial of a new form of dialysis
- Explore patients’ experiences of receiving incremental HD
- Gauge participants’ feelings about the regime of increased monitoring and tests undertaken as part of incremental HD.

#### Eligibility and recruitment

All participants who have undergone incremental HD (in the interventional arm of this study) will be eligible for participation in this phase of the study. Full eligibility criteria are presented in Table 2.

**Table 2:** Eligibility criteria for the interview phase of the study

Inclusion criteria	Exclusion criteria
Previously received incremental HD in the interventional arm of the ENDURE study OR a close relative of someone who has previously receive incremental HD in this study Willing and able to comply with study requirements Able to give written informed consent	Decline participation in this sub-study Unable to comply with requirements of the interview process defined in this section

#### Sample size, interview procedure and analysis

We aim to conduct 10–14 semi-structured interviews (but interviews will continue until data saturation is reached) [44]. Interviews will be guided by a topic guide (see Table 3) designed to capture patient understandings and experiences of the intervention and feasibility trial.

**Table 3:** Topic guide for participant interviews

Questions	Prompts/clarifications
Looking back to a time when you hadn’t yet started dialysis (i.e. when you were still under follow-up at pre-dialysis clinic), what were your expectations from dialysis?	Did you have any prior concerns about starting dialysis? What changes were you expecting dialysis would bring to your lifestyle or living arrangements? What changes were you expecting dialysis would bring to your symptoms?
What did you think about being approached for participation in the study?	You were approached in …………. (clinical setting), by ………… (study personnel), how did you find the experience? Would you suggest we did anything differently when approaching participants for the study in future? What went well? What could be improved?
What were the main reasons you said yes to participation in the study?	Did you discuss the study with your family? Did you discuss the study with other health professionals?
Did you have any concerns at the start about participating in the study?	Do you think your concerns were adequately addressed? In hindsight, what do you think we could have been done differently to pre-empt and address these concerns?
What are the main things that you got from the study?	Do you think there have been any advantages to taking part in the study?
Did you experience any problems?	How did the problem affect you? How did the problem affect your family/carers? In hindsight, what do you think could have been done to avoid this problem?
What changes, if any, do you think we need to make to the dialysis programme (the ‘incremental dialysis’) itself?	In incremental dialysis, you initially receive treatment twice weekly at shorter durations than usual, then your treatment is increased gradually over 3 months. I would like to get your thoughts on this programme: When you first started dialysis, did you notice an immediate change in how you felt? Were your symptoms changing with increasing dialysis (for better or worse)? You are now on three-times weekly full-length dialysis. With hindsight, did you have any symptoms previously that you think could have been because of less frequent dialysis?
The study included several additional procedures and tests that are not offered routinely to dialysis patients (these included questionnaires and urine collections). How did you find completing those tests?	Anything to report for the 24-h urine collections? Anything to report for the quality of life questionnaires? In future studies, should these tests be conducted more often, less often or at the same number of times as in this study?
Would you suggest we did anything differently in this study?	What changes do you think we need to make to the study overall to improve the incremental dialysis regime?
Would you recommend the study to other patients in future?	What advice would you give to someone who is about to start dialysis treatment and is offered a chance to start dialysis incrementally as part of a research study

All interviews will be conducted either face-to-face whilst patients receive their scheduled dialysis or over the internet video conferencing. Interviews will be conducted by a single researcher and are anticipated to last approximately 1 h.

With participants’ permission, the interviews will be recorded on an encrypted laptop or audio recording device which will then be transcribed verbatim and managed using Nvivo (QSR International, Australia). The transcripts will be anonymised, and all names will be replaced by a unique identifiers (IDs). Place or staff identifiers within the transcripts will be replaced by unit ID or staff job title. The study investigator will use inductive thematic analysis [45] to code the interview transcripts and then clustered into themes, along with anonymised quotes to support the account, will be presented in the final report.

### Study timeline and future plans

Final analyses will be completed after the 20th participant (or the last participant recruited within the 18-month recruitment period) has completed 6 months follow-up (September 2021). Data obtained from this feasibility study will be used to design the incremental HD regime to be used in a future RCT.

### Approvals and registration

This study has been approved by the West of Scotland Research Ethics committee-4 (Ref: 19/WS/0019). The protocol was registered on clinicaltrials.gov in February 2020 (NCT04268264).

## Discussion

### Statement of novelty

We have proposed a novel and pragmatic study that simplifies incremental HD by focusing on key elements of patient welfare and safety using commonly used measures in the care of dialysis patients.

There is currently no agreement on the optimal method of implementing incremental HD and the term ‘incremental HD’ has been used variably in the past to describe twice-weekly treatment regimens of different aims and degrees of complexity [46–49]. In one version described by Kaja Kamal et al. [48], twice-weekly dialysis is administered on an ongoing basis as long as RRF is maintained above stdKt/V_urea_ > 2. This requires regular RRF measurements through timed urine collections and clinicians to react to changes in RRF and adjust dialysis prescriptions. Kaja Kamal et al. have previously reported that the patients spend a median of 9 months receiving incremental HD before transitioning to three-times weekly HD [48]. A trial of this regime is currently underway [50].

Our version of incremental HD is novel as we apply twice-weekly HD in a time-limited manner (i.e., the first 15 weeks of dialysis), which does not require time urine collections. In our regime, the key aim is to focus on reducing the risk of decompensation in the early days of dialysis, while patients are still adapting (and hence aiming to reduce the high rates of early mortality) which we hypothesise is related to intensity of dialysis [51]—our approach is in contrast to other therapy regimens [19, 46, 52] where the primary aim of treatment is to preserve RRF and hence influencing medium/long-term outcomes. Our regime is of a relatively short duration as we expect RRF to decline significantly [53, 54] in first few months of dialysis making twice-weekly HD unsustainable.

Other key features that distinguish this version of incremental HD from other solutions proposed previously[46, 50, 55, 56] include: simplified eligibility criteria (see Table 1) that pre-selects out-patient HD starters who are well prepared for dialysis; lack of adequate preparation [22, 57] significantly carry worse prognoses and such patients are not suitable for incremental HD. Note that previous studies have used more complex screening methods such as timed urine collections at baseline to select suitable candidates for incremental HD, but concerns have been voiced about completion and accuracy of urine collections in dialysis patients [58–60].

As above, we have also bypassed the need for timed urine collections in the follow-up period by introducing monitoring measures that focus on key safety parameters (i.e. additional tests for hyperkalaemia and hydration status). This approach opens incremental HD to a wider section of new dialysis starters, particularly to the elderly. We pre-specify fixed increments in HD treatment times, providing more certainty to patients and helping dialysis staff with the planning of treatments.

The use historical controls is novel in dialysis research. A control arm is needed to compare the rates of pre-defined safety events, and to study the impact of incremental HD on selected patient outcomes. The delivery of modern HD therapy is highly regimented and treatment records are readily accessible through dedicated dialysis databases. This makes the use of historical controls a feasible option, particularly when examining rates of well-recognised events such as changes in pre-dialysis weights, BPs, episodes of hyperkalaemia and details of medical interventions. Our strategy hence represents a pragmatic approach which also increases the study power when comparing selected (the routinely collected) parameters [61, 62] and ensures timely completion of the study provided there is appropriate matching of key confounding attributes [63].

To our knowledge, this is the first study exploring the views of patients receiving incremental HD through recognised qualitative research methods. Analysing patient experiences are an important aspect of gauging future demand for incremental HD and forms key part of the feasibility works conducted here. The qualitative methods in this study will also aid in achieving a deeper understanding behind some of the quantitative findings (e.g., dropout rates) making these findings much more informative.

### Future implications

The results from this study will address a significant knowledge gap in the prescription of HD therapy and inform the development of novel therapy regimes in future. For patients, it would represent a less physiologically challenging transition into dialysis dependency which could reduce rates of early mortality and cardiovascular events, and possibly improve quality of life. Successful implementation of incremental HD could lead to substantial cost-savings to healthcare providers by reducing the number of treatments required at the start and hospitalisation episodes from complications of HD therapy. We believe that an RCT of incremental HD can be conducted within the next 5 years.

## Supplementary Information

Below is the link to the electronic supplementary material.

Supplementary file1 (DOCX 20 KB)

Supplementary file2 (DOCX 17 KB)
