# Supplementary material for: A mixed-method feasibility study of a novel transitional regime of incremental haemodialysis: study design and protocol
Source: Clin Exp Nephrol. 2021 Jun 8;25(10):1131–41. doi: 10.1007/s10157-021-02072-1 (PMC8421284; doi:10.1007/s10157-021-02072-1)
Supplement: Supplementary file 1 — Supplementary file1 (DOCX 20 KB) [file 10157_2021_2072_MOESM1_ESM.docx]

**Supplementary table S1:** Overview of study procedures

|  | **Scrn.** | **Base.** | **Treatment and follow-up** | | | | | |
| --- | --- | --- | --- | --- | --- | --- | --- | --- |
| **Visit Number** | **1** | **2** | **3** | **4** | **5** | **6** | **7** | **8** |
| **Procedures** | **Pre-HD** | **First HD**  **(Day 1)** | **Fourth HD**  **(Day 6-8)** | **Fifth HD**  **(Day 9-11)** | **Sixth HD**  **(Day 13-15)** | **1 month** | **3 months** | **6 months** |
| Informed consent | X |  |  |  |  |  |  |  |
| Demographics | X |  |  |  |  |  |  |  |
| Inclusion/Exclusion Criteria | X |  |  |  |  |  |  |  |
| Primary Diagnosis | X |  |  |  |  |  |  |  |
| Detailed History and Co-morbidities | X |  |  |  |  |  |  |  |
| Physical Examination |  | X | X |  | X | X | X |  |
| Weight (Kg) and Body mass index (Kg/m2) | X | X | X | X | X | X | X | X |
| Blood pressure (mmHg) |  | X | X | X | X | X | X | X |
| Medication check |  | X |  |  |  | X | X | X |
| Bio-impedance measure |  | X | X |  | X | X | X | X |
| **ROUTINE – NHS** |  |  |  |  |  |  |  |  |
| Biochemical profile/eGFR |  | X |  |  |  | X | X | X |
| Full blood count |  | X |  |  |  | X | X | X |
| Parathyroid hormone (ng/L) |  | X |  |  |  |  | X | X |
| Serum Ferritin (µg/L) |  | X |  |  |  |  | X | X |
| **NON – ROUTINE** |  |  |  |  |  |  |  |  |
| Urea and electrolytes (U&E), for additional potassium checks |  |  | X | X | X |  |  |  |
| C-reactive protein (mg/L) |  | X |  |  |  | X | X | X |
| N-terminal Pro-BNP (ng/L) |  | X |  |  |  | X | X | X |
| Transferrin Saturation (%) |  | X |  |  |  | X | X | X |
| Cystatin C (mg/dL) |  | X |  |  |  | X | X | X |
| Interdialytic urine collections |  | X |  |  |  | X | X | X |
| **Patient Assessments** |  |  |  |  |  |  |  |  |
| KDQOL-SF36 survey |  | X |  |  |  |  | X | X |
| Karnofsky Score |  | X |  |  |  |  | X | X |
| Charlson comorbidity Index |  | X |  |  |  |  | X | X |
