# Supplementary material for: A mixed-method feasibility study of a novel transitional regime of incremental haemodialysis: study design and protocol
Source: Clin Exp Nephrol. 2021 Jun 8;25(10):1131–41. doi: 10.1007/s10157-021-02072-1 (PMC8421284; doi:10.1007/s10157-021-02072-1)
Supplement: Supplementary file 2 — Supplementary file2 (DOCX 17 KB) [file 10157_2021_2072_MOESM2_ESM.docx]

**Supplementary table S2**: Adverse events (AE) and Serious adverse events (SAE) definitions.

| **Code** | **Name** |
| --- | --- |
| SAE 1 | Death from any cause |
| SAE 2 | Major adverse cardiovascular events (4p-MACE: CV death, nonfatal MI, nonfatal stroke, hospitalization for unstable angina) |
| SAE 3 | Hospitalisation |
| SAE 4 | Prolongation of existing hospitalisation |
| SAE 5 | Leads to permanent disability |
| AE 1 | Infections (if leads to hospitalisation) |
| AE 2 | Infections treated as outpatients: any antibiotic treatment in the first 6 months of starting HD |
| AE 3 | Intra-dialytic hypotension: Any drops in BP of > 20 mmHg systolic associated with nursing interventions (e.g. stopping ultrafiltration, lying the patient supine, or fluid bolus) |
| AE 4 | Access problem 1: complete loss of access which cannot be used for dialysis |
| AE 5 | Access problem 2: fistula required intervention |
| AE 6 | Access problem 3: fistula required resting (for any reason) |
| AE 7 | Hyperkalaemia 1:  Any pre-HD > 6.5 |
| AE 8 | Hyperkalaemia 2:  two consecutive pre HD K > 5.5 |
| AE 9 | Severe hypertension:  Pre HD SBP > 180 or DBP > 110 |
| AE 10 | Inter-dialytic weight gain > 4 KG |
| AE 11 | Missed planned dialysis session for any reason |
| AE 12 | Any other event which investigator believes may be linked to patient participation in the study |

CV, cardiovascular; MI, myocardial infarction; HD, haemodialysis/haemodiafiltration; K, potassium; BP, blood pressure; SBP, systolic blood pressure; DBP, diastolic blood pressure.
